# Experimental Assessment of Recycled Diesel Spill-Contaminated Domestic Wastewater Treated by Reed Beds for Irrigation of Sweet Peppers

**DOI:** 10.3390/ijerph13020208

**Published:** 2016-02-06

**Authors:** Suhad A.A.A.N. Almuktar, Miklas Scholz

**Affiliations:** Civil Engineering Research Group, School of Computing, Science and Engineering, The University of Salford, Greater Manchester, Salford M5 4WT, UK; s.a.a.a.n.almuktar@edu.salford.ac.uk

**Keywords:** *Capsicum annuum*, hydrocarbon, marketable yield, nutrient, sustainable agricultural water resource, water reclamation, wetland, vegetable

## Abstract

The aim of this experimental study is to assess if urban wastewater treated by ten different greenhouse-based sustainable wetland systems can be recycled to irrigate *Capsicum annuum* L. (Sweet Pepper; California Wonder) commercially grown either in compost or sand within a laboratory environment. The design variables were aggregate diameter, contact time, resting time and chemical oxygen demand. The key objectives were to assess: (i) the suitability of different treated (recycled) wastewaters for irrigation; (ii) response of peppers in terms of growth when using recycled wastewater subject to different growth media and hydrocarbon contamination; and (iii) the economic viability of different experimental set-ups in terms of marketable yield. Ortho-phosphate-phosphorus, ammonia-nitrogen, potassium and manganese concentrations in the irrigation water considerably exceeded the corresponding water quality thresholds. A high yield in terms of economic return (marketable yield expressed in monetary value) was linked to raw wastewater and an organic growth medium, while the plants grown in organic medium and wetlands of large aggregate size, high contact and resting times, diesel-spill contamination and low inflow loading rate produced the best fruits in terms of their dimensions and fresh weights, indicating the role of diesel in reducing too high nitrogen concentrations.

## 1. Introduction

### 1.1. Background and Motivation

Since water resources are limited in dry climates, sustainable wastewater treatment and the recycling of the corresponding effluent is a good alternative to using potable water for irrigation [1]. Treated urban water can be recycled for irrigation in agriculture, urban landscape management, industry and ground water recharge [2]. Around 20M ha of land is irrigated by untreated and treated wastewater [3]. Advantages linked to urban wastewater recycling include the abundant supply of nutrients to crops, which could lead to higher marketable yields as well as a decrease in the need for commercial fertilizers [4].

### 1.2. Wastewater Recycling for Irrigation

Wastewaters can be treated with a wide range of standard (e.g., activated sludge process or trickling filters) and alternative (e.g., wetlands) technologies that can be selected based on criteria such as reliability, simplicity, efficiency, land requirement, affordability, social acceptability and sustainability [5]. Various practical and academic tools have been developed to assist decision-makers in selecting the most appropriate technology including expert opinion [6,7].

Treated wastewater can be recycled to irrigate crops in arid regions that are confronted by considerable water shortages, supporting renewable agriculture and food systems. However, pre-treated wastewaters might require disinfection before application to fields [6]. Irrigation by recycled wastewater can increase the productivity of farming by between 100% and 400%, allowing some crops to be grown in regions with unfavorable conditions [8].

The assessment of the impacts of wastewater reuse on agricultural products intended for consumption is important [9,10,11]. Either too high or too low concentrations of nutrients in the reused water are a potential problem to crops. Hydrocarbons within road runoff are a new challenge [12,13].

Researchers [12] studied the impact of treated urban wastewater on soil and Sweet Pepper. Recycling saved fertilizer. Moreover, hydrocarbons and heavy metals were low in the harvested fruits. The reuse of nutrients from settled primary urban wastewater on peppers was researched, previously [14]. The crops grown removed both nitrogen and phosphorous, and were healthy compared to the control using standard fertilizer. Nutrients such as ammonia have a negative effect on fruit, leaf and stem developments [15]. Moreover, the total yield increases as the nitrate-nitrogen to ammonia-nitrogen ratio increases. This can be explained by a reduction in fruit physiological disorders, which usually reduce fruit mean weight [16].

Research was undertaken to evaluate the impact of various growth media on Sweet Pepper yields, indicating that peat moss media benefited seedlings. Moreover, peat moss and coco-peat alone or mixed with sand led to a better harvest than other media [17]. The yield response of peppers to mineral and organic fertilization has been assessed, previously [18]. Findings indicate that no differences in yield were noted between organic and conventional farming practices. Another study was undertaken to assess the effects of only peat, and a mixture of peat, perlite and sand (volume ratio of 1:1:1) on yield-related parameters. Results indicated that mixed media increased length, diameter and weight of fruits in all cultivars in comparison to only peat media [19].

Wetland systems can be applied to treat urban wastewater well [13,20]. The treatment efficiency of constructed wetlands on wastewaters and their suitability for reuse in agriculture was also assessed elsewhere [21]. Only water quality variables with high removal efficiencies fulfilled the guidelines for recycling. However, parameters with rather low efficiencies such as solids and phosphorus limited the water reuse potential. Wetlands treating urban effluents to be reused in the agricultural sector were assessed previously [22]. Mean removal efficiencies for suspended solids, biochemical oxygen demand, chemical oxygen demand, total nitrogen and total phosphorus were 85%, 65%, 75%, 42% and 32%, respectively, indicating the possibility for ecological sanitation.

### 1.3. Nutrient and Mineral Requirements

The authors of this article focused on Sweet Pepper, because it is an easy-to-grow and cost-effective plant with good nutritional benefits [23]. The major elements influencing the growth of crops are nitrogen, phosphorus, potassium, calcium, magnesium and sulphur [24]. Heavy metals may be toxic for peppers.

Maximum concentrations for iron, manganese and potassium are 5.0 mg/L, 0.2 mg/L and 2.0 mg/L, respectively [25,26]. The application of various treated wastewaters for recycling has been classified [27]. Suitable ranges for ammonia-nitrogen and ortho-phosphate-phosphorous are 0 mg/L to 5 mg/L, and 0 mg/L to 2 mg/L in that order. Furthermore, no restriction for the reuse of pre-treated waters with a nitrate-nitrogen value below 5 mg/L has been proposed. Slight to moderate constraints exist for the range between 5 mg/L and 30 mg/L. However, severe restrictions are imposed for measurements greater than 30.0 mg/L [26].

### 1.4. Rationale, Aim and Objectives

Effluents from different wetlands treating urban wastewater were recycled to irrigate Sweet Pepper. Some wetlands received wastewater contaminated by diesel. The aim of this study is to evaluate if Sweet Pepper can be grown successfully using recycled urban wastewater treated by wetlands to obtain a high marketable yield.

The specific and measurable objectives related to the growing of peppers are to assess: (a) the appropriateness of treated (recycled) wastewater for irrigation compared to corresponding standards; (b) the impact of various waters as a function of the type of wetland; (c) the response of peppers in terms of growth when using recycled (pre-treated) wastewater streams (some contaminated by a diesel spill) subject to different growth media; (d) the effect of environmental boundary conditions on the yield; and (e) the economic return of various experimental systems in terms of marketable yield.

## 2. Materials and Methods

### 2.1. Wetlands Set-Up and Operational Arrangements

Ten vertical-flow wetland filters were operated between 27 June 2011 and 25 September 2014. The design variables were aggregate diameter, contact time, resting time and chemical oxygen demand. Contact time is defined as the period of time when the inflow water stays within the wetland. In comparison, resting time indicates the duration when the wetland is drained. The set-up includes two controls receiving clean de-chlorinated tap water (Table 1).

**Table 1:** Comparison of the experimental vertical-flow wetland set-up.

Filters ^a^	Design variables			
	Aggregate Diameter (mm)	Contact Time (h)	Resting Time (h)	Chemical Oxygen Demand (mg/L)
Filter 1 ^b^	20	72	48	123.3
Filter 2	20	72	48	123.3
Filter 3 ^b^	10	72	48	123.3
Filter 4	10	72	48	123.3
Filter 5 ^b^	10	72	48	244.7
Filter 6	10	72	48	244.7
Filter 7	10	36	48	123.3
Filter 8	10	36	24	123.3
Control A ^b^	10	72	48	2.3
Control B	10	72	48	2.3

**^a^** Annually treated volumes of wastewater: Filters 1 to 6, 470 L/a; Filter 7, 624 L/a; Filter 8, 858 L/a; Control A and B, 470 L/a. ^b^ On 26 September 2013, 130 g of diesel (equivalent to an inflow concentration of 20 g/L) have been added to Filters 1, 3 and 5, and Control A.

The wetland filters were constructed from Pyrex tubes (inner diameter of 19.5 cm and height of 120 cm). The filters were filled with pea gravel up to 60 cm and planted with *Phragmites australis* (Cav.) Trin. ex Steud. (Common Reed)(*P. australis*). The outlet valve is located at the bottom of each filter [28].

Preliminary treated wastewater was obtained from the Davyhulme Sewage works in Manchester. In order to simulate diesel fuel spills, 130 gram of diesel were poured into Filters 1, 3 and 5, and into Control A on 26 September 2013. Chillers (Aquacadabra, Barnehurst Road, Bexleyheath, UK) were used to maintain the root system at 12 °C. All wetland columns received approximately 6.5 L of inflow [28]. All water quality parameters were recorded during or directly after harvesting the wastewater from the wetland filter.

### 2.2. Water, Soil and Pepper Quality Analysis

Routine water quality sampling was carried out according to standard methods [29]. The analysis of water samples for nutrients and trace element concentrations was undertaken using Varian 720-ES Inductively Coupled Plasma—Optical Emission Spectrometer technology (ICP–OES [30]) manufactured by Agilent Technologies UK (Wharfedale Road, Wokingham, Berkshire, UK).

Soil quality analysis was undertaken [31]. Pepper marketable yield was assessed according to Table 2 [32,33,34]. Microbial tests (total coliforms, *Escherichia coli*, fecal *Streptococcus* spp. and *Salmonella* spp.) for water and vegetables (skin, flesh and washing solution harvested at different distances from the soil: 0 to 50 cm, 50 to 100 cm and more than 100 cm) were performed using aseptic pour plate techniques according to standard methods [29]. Where relevant, sample numbers, sampling periods and replicate numbers for water, soil and plant samples have been identified in the illustrations shown in Section 3.

### 2.3. Light, Humidity and Temperature

Light measurements were undertaken using the LUX meter ATP-DT-1300 (TIMSTAR, Road Three, Winsford Industrial Estate, Winsford, Cheshire, UK). Humidity and temperature were recorded using wetterladen24.de (JM Handelspunkt, Geschwend, Germany). The humidity was controlled using Challenge 3.0L Ultrasonic Humidifiers (Argos, Avebury Boulevard, Central Milton Keynes, England, UK).

### 2.4. Sweet Pepper Growing

Sweet Pepper seeds were obtained from B&Q plc (Chandlers Ford, Hants SO53 3LE;) on 14 September 2013. The seeds were first planted in shallow seed trays for about one week, and subsequently replanted (second planting) in larger nursery pots. The third and final planting was undertaken 28 days after the second planting on 8 November 2013, following supplier instructions. The peppers were planted into 10-litre plastic and round plant pots sourced from scotplants (Hedgehogs Nursery, Crompton Road, Glenrothes, Scotland, UK).

Plant pots dimensions were: height of 22.0 cm, bottom diameter of 22.0 cm and top diameter of 28.5 cm. Compost and pure sand were used. The compost was supplied by B&Q plc as part of their verve brand (product code: 03717644). The sand (Play Pit Sand (silica), product code: 5060096123309) was provided by Deko-Pak Limited (Deco House, Halifax Road, Hipperholme, Brighouse HX3 8BW).

The basic soil properties are listed in Table 3. The top 2 cm were left unplanted for both sand- and soil-based pots. However, sand-based plants were planted to a depth of 20.0 cm, and soil-based plants were planted to a depth of 17.5 cm, and covered by 2.5 cm of bark (B&Q verve range, product code: 5397007188110), which was described by B&Q as ideal for pots, beds and borders to control weeds, retain moisture and insulate soil. The plant pots remained indoors under laboratory conditions characterized in Section 2.3.

Some peppers received fertilizer sourced from the B&Q plc verve range (product code: 5397007068245). The fertilizer had a nitrogen to phosphorus to potassium ratio of 4:4:4. Liquid fertilizer was added to the inflow water as specified.

### 2.5. Data Analysis

Microsoft Excel and IBM SPSS Statistics Version 20 were used. Significant findings have been highlighted, where appropriate.

**Table 2:** Sweet Pepper harvest classification scheme (partly adopted from elsewhere [32,33,34].

Variable	Class A	Class B	Class C	Class D	Class E
Quality class	Outstanding	Good	Good	Satisfactory	Unsatisfactory
European Union classification equivalent	“Extra” Class	Class I	Class II	Not applicable	Not applicable
Mean price estimate; pence (Sterling)/gram	0.28	0.22	0.16	0.10	0.00
Target market	Top restaurant	National supermarket	Independent retailer or market	Vegetable industry	Waste company
Product	Fresh vegetable	Fresh vegetable	Fresh vegetable	Frozen or canned	Waste
Contamination	Uncontaminated	Uncontaminated	Uncontaminated	Uncontaminated	Contaminated
Illnesses	None	None	None	Likely; no harm	Likely; harmful (rotten)
Length (L, mm)	Jumbo (L ≥ 110)	Extra-large (90 ≤ L < 110)	Large (70 ≤ L < 90)	Medium (40 ≤ L < 70)	Small (L < 40)
Diameter (D, mm)	Jumbo (D ≥ 90)	Extra-large (70 ≤ D < 90)	Large (50 ≤ D < 70)	Medium (30 ≤ D < 50)	Small (D < 30)
Weight (w, g)	Very Large (w ≥ 190)	Large (120 ≤ w < 190)	Medium (70 ≤ w ≤ 120)	Small (20 ≤ w < 70)	Very Small (w < 20)
Tolerance by weight or number per plant (%)	5	10	10	10	10
Defect in shape (Damage (%) of surface area)	Damage ≤ 10	10 ≤ Damage < 20	20 ≤ Damage < 30	30 ≤ Damage < 60	Too many damages (>60)
Defect of the skin (Damage (%) of surface area)	Damage ≤ 3	3 ≤ Damage < 4	4 ≤ Damage < 5	5 ≤ Damage < 20	Too many damages (>20)

**Table 3:** Basic soil properties based on three replicates each (14 September 2013).

Parameter	Soil Type		Total Per Pot (mg)	
	Compost	Sand	Compost	Sand
pH	6.43	9.40	-	-
Redox potential (mV)	62.60	−79.20	-	-
Electrical conductivity (μs/cm)	2438.50	116.00	-	-
Total nitrogen (mg/kg)	998.75	7.60	3495.63	114.00
Total phosphor (mg/kg)	367.50	0.85	1286.25	12.75
Aluminium (mg/kg)	1118.38	1180.43	3914.33	17,706.45
Calcium (mg/kg)	18,421.96	174.16	64,476.86	2612.40
Iron (mg/kg)	6233.15	1196.48	21,816.03	17,947.20
Potassium (mg/kg)	2776.02	168.57	9716.07	2528.55
Magnesium (mg/kg)	5287.67	279.53	18,506.85	4192.95
Manganese (mg/kg)	201.59	8.09	705.57	121.35
Zinc (mg/kg)	26.59	1.95	93.07	29.25
Boron (mg/kg)	12.29	<0.0001	43.02	0.0015
Organic matter (%)	89.00	0.03	-	-
Bulk density(g/L)	350	1522	-	-

## 3. Results and Discussion

### 3.1. Comparison of Irrigation Water Qualities

#### 3.1.1. Comparison of Hydrocarbon Values

Table 4 shows the inflow water quality. Total petroleum hydrocarbon values followed this order: Control A > Filter 8 > Filter 1 > Filter 3> Filter 5. Regarding filters contaminated by diesel (Filters 1, 3, 5 and Control A), the total petroleum hydrocarbon concentrations were 0.100 mg/L, 0.069 mg/L, 0.014 mg/L and 0.346 mg/L, respectively. These concentrations were in compliance with Chinese standards [35], indicating a maximum threshold of 1.0 mg/L. Note that Chinese standards were used, considering that China produces about 54% (estimated in 2008) of peppers in the world [36].

#### 3.1.2. Comparison of Oxygen Demand Variables

Table 4 shows that chemical oxygen demand values were the highest for raw urban wastewater followed by filters contaminated with diesel following the order of F5 > F3 > F1 > Control A. Chemical oxygen demand concentrations were highly variable due to seasonal changes. Moreover, the standard deviation for Filter 8 was particularly high due to the low resting time (Table 1) resulting in insufficient biodegradation in some seasons. Statistically, no significant difference (*p* > 0.05) in chemical oxygen demand values of Filters 2 and 4 were found, indicating that aggregate size may not matter (Table 1). Filter 8 outflow water had chemical oxygen demand values, which were higher than those of Filter 7, highlighting the impact of long resting time on outflow water chemical oxygen demand (Table 1). In comparison, the lowest chemical oxygen demand values were recorded for Control B (no diesel contamination; Table 1).

The biochemical oxygen demand was the highest for raw urban wastewater and corresponding samples diluted with dechlorinated potable water followed by filters contaminated with diesel (Filters 1, 3 and 5). The biochemical oxygen demand values for Filter 8 (short resting time) outflow water were higher than those for Filter 7 (long resting time). The corresponding values for Control A (contaminated with diesel) were higher than those for Control B. Tap water had the lowest biochemical oxygen demand (Table 4).

**Table 4:** Comparison of the water quality of the inflow waters received by the vegetable pots (mean ± standard deviation (number of samples)) between 11 October 2013 and 25 September 2014.

Water Type	TPH ^a^ (µg/L)	COD ^b^ (mg/L)	BOD ^c^ (mg/L)	NH_4_-N ^d^ (mg/L)	NO_3_-N ^e^ (mg/L)	PO_4_-P ^f^ (mg/L)	SS ^g^ (mg/L)	Turbidity (NTU) ^h^	Ph (−)	EC ^I^ (µS/cm)	DO ^j^ (mg/L)	SAR ^k^ (me/L) ^l^
Filter 1 outflow	100	77.7 ± 23.35 (18)	25.8 ± 16.74 (53)	4.8 ± 2.83 (22)	0.4 ± 0.22 (19)	4.0 ± 2.48 (18)	11.3 ± 10.42 (56)	9.0 ± 5.65 (54)	6.4 ± 0.26 (54)	336.5 ± 50.82 (22)	1.5 ± 1.03 (15)	2.4 ± 1.07 (5)
Filter 2 outflow	<10	34.9 ± 19.21 (15)	13.6 ± 8.11 (51)	6.2 ± 5.84 (20)	2.2 ± 2.72 (18)	3.3 ± 1.33 (18)	6.7 ± 9.49 (56)	5.4 ± 5.75 (53)	6.5 ± 0.21 (54)	328.6 ± 53.37 (22)	1.7 ± 1.10 (15)	1.8 ± 0.60 (5)
Filter 3 outflow	69	87.5 ± 26.00 (18)	22.8 ± 16.42 (51)	3.7 ± 2.53 (22)	0.4 ± 0.28 (19)	3.3 ± 2.04 (18)	11.7 ± 10.79 (56)	8.7 ± 6.09 (53)	6.5 ± 0.18 (54)	396.7 ± 76.59 (22)	1.7 ± 1.18 (15)	1.7 ± 0.53 (5)
Filter 4 outflow	<10	34.9 ± 23.77 (15)	12.8 ± .8.86 (50)	5.0 ± 10.53 (20)	1.8 ± 3.27 (18)	2.9 ± 1.06 (18)	7.4 ± 10.57 (56)	5.7 ± 5.46 (53)	6.5 ± 0.19 (54)	352.6 ± 67.56 (22)	2.0 ± 1.60 (15)	2.4 ± 0.39 (5)
Filter 5 outflow	14	100.8 ± 67.90 (18)	22.5 ± 16.35 (51)	9.7 ± 3.20 (21)	0.9 ± 0.86 (19)	4.4 ± 2.07 (18)	11.3 ± 12.76 (57)	8.6 ± 6.22 (53)	6.6 ± 0.19 (54)	564.1 ± 163.66 (22)	1.5 ± 0.81 (15)	2.2 ± 0.85 (5)
Filter 6 outflow	<10	35.6 ± 22.46 (14)	15.9 ± 12.68 (52)	9.0 ± 7.28 (20)	3.6 ± 4.68 (18)	4.6 ± 3.16 (18)	6.9 ± 8.68 (57)	5.4 ± 4.41 (53)	6.8 ± 0.19 (55)	524.3 ± 152.66 (22)	1.6 ± 1.09 (15)	3.1 ± 0.61 (5)
Filter 7 outflow	<10	32.5 ± 20.40 (14)	11.9 ± 8.01 (61)	3.6 ± 5.52 (24)	2.8 ± 2.98 (18)	3.6 ± 2.23 (17)	2.6 ± 3.86 (66)	3.4 ± 2.24 (62)	6.6 ± 0.18 (62)	355.0 ± 83.11 (28)	1.7 ± 0.86 (25)	2.4 ± 0.47 (5)
Filter 8 outflow	116	55.9 ± 86.05 (15)	13.9 ± 7.50 (69)	1.4 ± 1.35 (22)	2.8 ± 3.51 (16)	3.3 ± 1.90 (16)	2.9 ± 4.31 (76)	3.6 ± 2.48 (76)	6.5 ± 0.20 (78)	339.7 ± 104.74 (25)	1.9 ± 1.15 (22)	2.1 ± 0.44 (5)
Control A outflow	346	66.4 ± 44.32 (17)	12.0 ± 7.58 (51)	1.3 ± 1.79 (22)	0.4 ± 0.44 (19)	1.8 ± 0.56 (18)	9.0 ± 10.25 (56)	5.7 ± 4.31 (53)	6.7 ± 0.17 (55)	149.2 ± 32.47 (22)	1.4 ± 0.93 (15)	0.5 ± 0.15 (5)
Control B outflow	<10	16.0 ± 15.12 (15)	8.8 ± 7.58 (52)	1.3 ± 1.77 (21)	0.3 ± 0.35 (18)	1.9 ± 0.33 (18)	3.6 ± 8.18 (56)	4.1 ± 4.54 (53)	6.5 ± 0.20 (54)	153.9 ± 29.87 (22)	1.8 ± 1.04 (15)	0.5 ± 0.14 (5)
Deionised water	Nm ^m^	3.5 ± 0.08 (3)	7.3 ± 1.84 (3)	0.1 ± 0.13 (3)	0.0 ± 0.00 (3)	0.0 ± 0.00 (3)	2.0 ± 2.31 (10)	1.3 ± 0.14 (10)	5.1 ± 0.58 (10)	1.5 ± 0.72 (10)	nm	0.1 ± 0.15 (5)
Tap water (100%)	Nm	6.2 ± 0.33 (3)	4.9 ± 1.13 (3)	0.1 ± 0.00 (3)	0.2 ± 0.00 (3)	0.8 ± 0.00 (3)	2.0 ± 2.31 (10)	1.4 ± 0.21 (10)	6.1 ± 1.06 (10)	95.8 ± 15.20 (10)	nm	0.8 ± 0.15 (5)
Tap water with fertiliser	Nm	8.6 ± 0.22 (3)	8.0 ± 2.62 (3)	16.0 ± 0.01 (3)	8.9 ± 0.38 (3)	14.9 ± 0.07 (3)	1.6 ± 0.46 (10)	3.0 ± 0.49 (10)	6.0 ± 0.28 (10)	204.0 ± 5.66 (10)	nm	0.8 ± 0.10 (5)
Wastewater (20%); tap water (80%)	Nm	47.6 ± 15.39 (17)	21.8 ± 15.99 (55)	6.7 ± 3.69 (22)	0.5 ± 0.64 (21)	3.0 ± 1.43 (21)	26.4 ± 18.48 (63)	16.2 ± 15.18 (56)	7.1 ± 0.07 (55)	122.1 ± 55.98 (22)	nm	1.7 ± 0.59 (5)
Wastewater (100%)	Nm	237.9 ± 76.96 (17)	105.3 ± 75.98 (55)	33.6 ± 18.46 (22)	2.4 ± 3.22 (21)	14.9 ± 7.15 (21)	131.9 ± 92.64 (63)	80.4 ± 75.97 (56)	7.5 ± 0.42 (55)	575.5 ± 181.66 (22)	5.2 ± 3.72 (16)	2.8 ± 0.62 (5)
Standard	1000	-	-	5	30	2			6.0–8.5	3000		≤15

^a^ TPH: total petroleum hydrocarbon; ^b^ COD: chemical oxygen demand; ^c^ BOD: five-day biochemical oxygen demand; ^d^ NH_4_-N: ammonia-nitrogen; ^e^ NO_3_-N: nitrate-nitrogen; ^f^ PO_4_-P: ortho-phosphate-phosphorus; ^g^ SS: suspended solids; ^h^ NTU: turbidity; ^i^ EC: electrical conductivity; ^j^ DO: dissolved oxygen; ^k^ SAR: sodium adsorption ratio (sodium (calcium+magnesium)^−2^)^−0.5^); ^l^ me/L: milliequivalent per litre; ^m^ nm: not measured.

Dissolved oxygen values were higher for those filters without diesel (Table 4). Correlation analysis results indicated that dissolved oxygen was significantly negatively correlated with micro-organisms, total petroleum hydrocarbon and chemical oxygen demand in the treatment system. This negative correlation can be explained by an improvement of the chemical oxygen demand and the total petroleum hydrocarbon removal efficiencies as micro-organisms responsible for biodegradation acclimatized, resulting in a reduction of the amount of available dissolved oxygen [13].

#### 3.1.3. Comparison of Nitrogen Compounds

The inflow water quality was highly variable for both nitrogen species, because of seasonal water quality variations in the wetland systems (Table 4). The inflow waters with a high chemical oxygen demand resulted in statistically significant (*p* < 0.05) differences between the ammonia-nitrogen concentrations of Filters 3 and 4 compared to those of Filters 5 and 6, respectively (Table 1). Elevated concentrations of ammonia-nitrogen exceeding the threshold of 5 mg/L [25,26] were recorded for raw wastewater, wastewater diluted with 80% of potable water, and outflow waters from Filters 5 and 6, which were fed with high inflow loads, followed by that from Filter 2, which had a large aggregate size (Table 4).

Moreover, the mean nitrate-nitrogen values of Filter 4 compared to those of Filters 7, and the concentrations for Filters 3 and 4 compared to those of Filters 5 and 6 were statistically significantly different from each other (*p* < 0.05), indicating the impact of contact time and inflow loading rate of wetland systems on outflow water nitrate-nitrogen values (Table 1). However, nitrate-nitrogen concentrations for all outflows (Table 4) were below 4 mg/L, which is considerably less than the threshold of 30 mg/L [25,26].

#### 3.1.4. Comparison of Ortho-Phosphate-Phosphorus

Considering the threshold of 2 mg/L for ortho-phosphate-phosphorus [25], the outflow waters from all wetlands were associated with too high ortho-phosphate-phosphorus concentrations (Table 4). Statistical results did not show any significant differences in ortho-phosphate-phosphorus values of the outflow waters indicating that wetland aggregate diameter, contact and resting times as well as inflow loading rate do not matter (Table 1). However, phosphorus is difficult to remove by mature wetlands [37], because it is often present in particulate form [13].

#### 3.1.5. Comparison of Trace Elements

**Figure 1:**
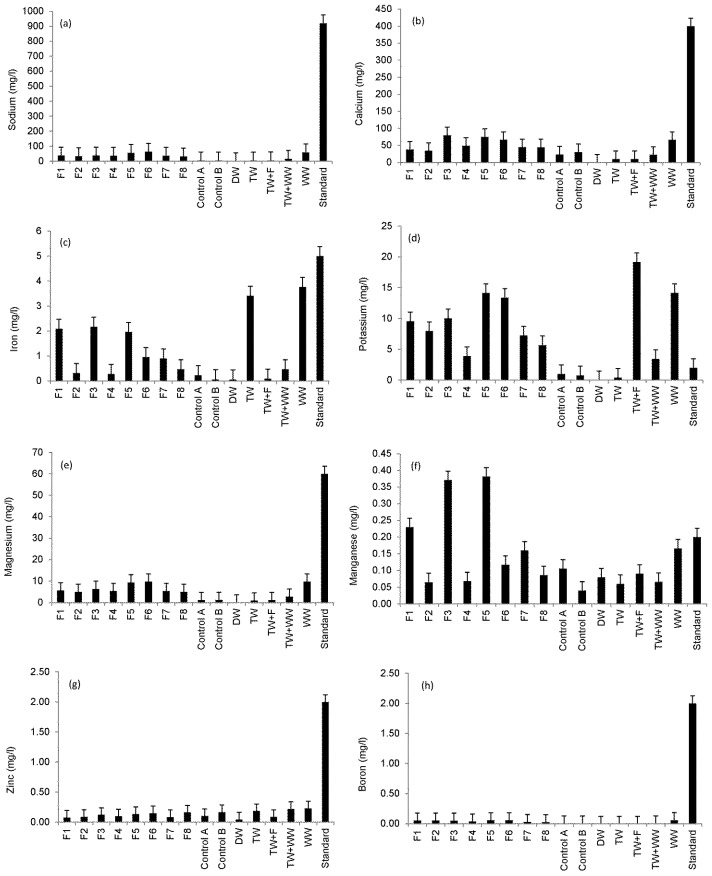
Overview of the Inductively Coupled Plasma—Optical Emission Spectrometer (ICP–OES) analysis (sample number: 10; 11 October 2013 to 25 September 2014) for detected elements compared with common standards for irrigation water [25,27]: (**a**) sodium; (**b**) calcium; (**c**) iron; (**d**) potassium; (**e**)~magnesium; (**f**) manganese; (**g**) zinc; and (**h**) boron.

Figure 1 shows an overview of the ICP–OES findings for selected elements determined in the irrigation water compared to standards. Figure 1a shows that sodium concentration for all irrigation waters did not exceed the standard for irrigation water of 920 mg/L [25,27]. However, statistical analysis showed that the sodium values of Filters 3 and 4 compared to those of Filters 5 and 6 outflow waters were significantly different from each other indicating the impact of the inflow loading rate of wetland systems on outflow water sodium concentrations (Table 1).

Figure 1b,c shows that the calcium and iron concentrations of all irrigation waters did not exceed the standards of 400 mg/L and 5 mg/L, respectively. Regarding the threshold of 2 mg/L for potassium [25,27], all irrigation water types (except for Controls A and B) had too high potassium concentrations. Statistical results showed that outflow water from Filter 4 had potassium concentrations, which were significantly different from those associated with Filter 7, indicating the impact of contact time on outflow water potassium concentrations (Table 1).

Moreover, a high inflow loading rate of the wetland system resulted in significant differences (*p* < 0.05) in outflow water potassium concentrations for Filter 4 compared with Filter 6 (Figure 1d). No magnesium concentrations exceeded the threshold of 60 mg/L [25,27] as shown in Figure 1e. However, statistical results showed that outflow waters from Filters 3 and 4 compared to those of Filters 5 and 6 had magnesium concentrations, which were significantly (*p* < 0.05) different from each other, explaining the impact of inflow loading rate of wetland systems on outflow water magnesium concentrations (Table 1). Figure 1f shows that results for Filters 1, 3 and 5, which were contaminated with diesel, have elevated manganese concentrations exceeding the threshold of 0.2 mg/L [25,27]. Figure 1g,h show that zinc and boron concentrations in all irrigation water types did not exceed the threshold of 2 mg/L [25,27].

#### 3.1.6. Comparison of Particles

The inflow water quality was highly variable for particles indicated by suspended solids and turbidity (Table 4). This can also be explained by the seasonal water quality variations in the wetland systems. For example, as above-ground *P. australis* plant parts decay in winter and early spring, more particles are created as by-products of the biodegradation process. Furthermore, the standard deviations for very clean waters such as tap water are high due to the random presence of larger particles.

Table 4 shows that the highest value for suspended solids was noted for raw wastewater and diluted wastewater followed by those for outflow waters from filters contaminated with diesel (Filters 1, 3 and 5, and Control A). Turbidity had the highest values for raw wastewater and diluted wastewater followed by outflow waters received from Filters 1, 3 and 5, which were contaminated with diesel. Filters 7 and 8 had the lowest turbidity values in spite of different resting times. Correlation analysis results showed that turbidity was significantly positively correlated with suspended solids and micro-organisms in treatment systems indicating a good relationship between turbidity and indicator micro-organisms activity due to degradation of organic matter and a subsequent increase in particles [28]. However, high values of suspended solids and turbidity associated with irrigation water will considerably increase the development of hydrophobicity in the soils, and subsequently affect plant growth.

#### 3.1.7. Comparison of pH and Salinity

Table 4 shows that the pH values were normal [25]. Conductivity is the most important indirect measure of salinity, posing a great hazard to crops and determining the suitability of water for irrigation use. Salts negatively impact on the growth of plants, and the soil structure and permeability, indirectly affecting plant growth as well. However, the conductivity values for all filter outflow waters complied with the threshold of 3000 μs/cm [25,27]. Furthermore, the sodium adsorption ratio concentrations for all outflows were normal; *i.e.*, between 0 milliequivalents per liter (me/L) and 15 me/L [27].

#### 3.1.8. Comparison of Microbial Content

**Figure 2:**
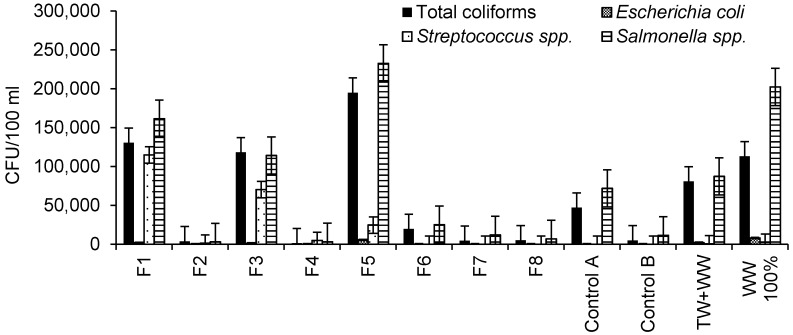
Microbiological characteristics of irrigation water (sample number: 20; 11 October 2013 to 25 September 2014).

Microbial characteristics of irrigation water are summarized in Figure 2. Based on the maximum value for total coliforms (1000 CFU per 100 mL) regarding the irrigation of crops [38], the outflow waters from all wetlands were associated with too high contamination by total coliforms. Furthermore, high contamination by total coliforms was also observed for raw wastewater and diluted wastewater.

Raw wastewater was associated with the highest contamination by *Escherichia coli* (8000 CFU per 100 mL) followed by outflow water from Filter 5 and wastewater. Outflow waters from Filters 1 and 3 had similar numbers of *Escherichia coli*. No contamination by *Escherichia coli* was detected for outflow waters from other wetlands.

The highest contamination by *Streptococcus* spp. was associated with Filter 1 outflow water followed by those for Filters 3 and 5, which were contaminated with hydrocarbons (Figure 2). Filter 4 had a higher *Streptococcus* spp. contamination than Filter 2, indicating the effect of aggregate size when comparing these filters with each other (Table 1). Furthermore, raw wastewater was more contaminated by *Streptococcus* spp. than diluted wastewater. The highest *Salmonella* spp. counting was observed in the outflow water from Filter 5 followed by raw wastewater (Figure 2). Filter 1 outflow water was associated with higher *Salmonella* spp. contamination than the water from Filter 3, highlighting the impact of aggregate size (Table 1). Furthermore, *Salmonella* spp. contamination in Control A outflow water was higher than that associated with Control B, explaining the effect of hydrocarbon contamination.

Figure 2 showed that the microbial contamination of outflow water from wetland filters polluted with hydrocarbon was higher than those from standard filters (uncontaminated). This confirms previous findings [39], assessing the impact of long-term total petroleum hydrocarbon (TPH) on the structure of bacterial communities. The results indicated that a high concentration of TPH positively impacted on the diversity of hydrocarbon-degrading bacteria. Furthermore, wetland filters fed with undiluted inflow water showed higher microbial contamination levels than those fed with diluted inflow, confirming other findings [28], indicating that high-rate filters tend to be overloaded.

### 3.2. Environment Boundary Conditions

Table 5 shows environmental boundary conditions. The light intensity records for this experiment during the flowering and fruiting stage were below the proposed range from about 8600 lux to 17,200 lux [40]. Low light intensity may lead to flower inhibition or cause flower abscission [41]. Moreover, low light intensity applied to plants will produce leggy plants growing toward light, which is necessary for photosynthesis [42].

For the germination stage (Table 5), the temperature records complied with the optimal temperature for peppers during this phase [43]. Concerning the vegetative growth stage, the temperature records (Table 5) for this experiment were higher than the recommended optimum values of between 21 °C and 23 °C [44]. However, temperature records for this stage complied with the values associated with the highest photosynthesis rate, which can be achieved at temperatures between 24 °C and 29 °C [45,46].

Table 5 shows that the relative humidity before and after fruiting was low (37 ± 7.6% and 57 ± 7.8%, respectively). Humidity values below 50% could have a negative impact on the fruit development as humid atmosphere is necessary for flowers to successfully pollinate; otherwise, the unfertilized flowers will drop off as reported elsewhere [46].

### 3.3. Sweet Pepper Growth Comparisons

**Figure 3:**
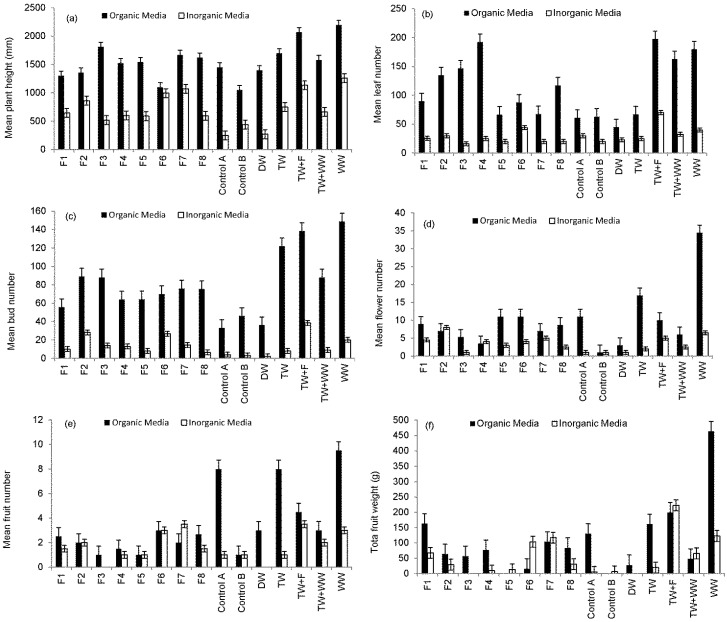
Comparison in growth of plants grown in different media and subjected to different irrigation water types (harvest between 20 January and 25 September 2014): (**a**) mean plant height; (**b**) mean leaf number; (**c**) mean bud number; (**d**) mean flower number; (**e**) mean fruit number; and (**f**) mean fruit weight.

Figure 3a,f shows a growth comparison between pepper plants growing in organic and inorganic media in terms of plant overall height, number of leaves, buds, flowers and total weight of fruits harvested from each treatment. Findings indicate that compost compared to sand is associated with considerably greater plant growth and productivity. This is due to the elevated nutrient availability in the basic compost [17] compared to sand (Table 3). Furthermore, organic substrate decomposes, releasing nutrients [47,48].

**Figure 4:**
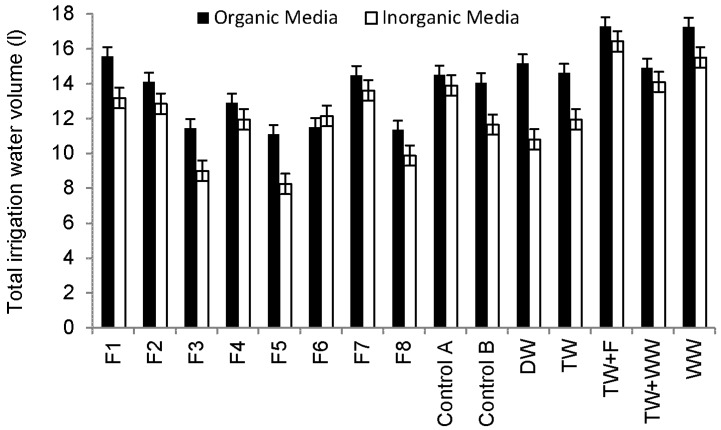
Overview of total irrigation water volumes for Sweet Pepper plants during the whole experiment period (11 October 2013 to 25 September 2014).

Sweet Pepper prefers light and well-drained soil, which is rich in organic substances with a pH value from 6.5 to 7.5 (Table 3) [43]. However, under acid soil conditions (soil pH < 7), heavy metals could be a challenge to Sweet Pepper [49]. Figure 4 shows that plants grown in compost consume more water than those grown in sand and subsequently increase the nutrient load applied to plants via irrigation water, leading to higher foliage and yield production.

Regarding the overall height of plants growing in organic media, Figure 3a shows that the maximum height was associated with plants irrigated with raw wastewater followed by those irrigated with tap water spiked with fertilizer. This can be explained by the high nutrient load (Table 6) applied via irrigation water. Results were statistically significantly different (*p* < 0.05) for the overall height of plants irrigated with water harvested from Filter 7 and Control B.

Regarding the total number of leaves (Figure 3b) linked to peppers grown in organic media, findings indicated that peppers irrigated with tap water spiked by fertilizer produced the highest leave number followed by those plants irrigated with water harvested from Filter 4 and raw wastewater, while the lowest leave numbers were recorded for plants irrigated with deionized water followed by tap water and Controls A and B.

**Table 5:** Overview of environmental boundary conditions associated with the vegetable pots (mean ± standard deviation (number of records)).

Parameter	Unit	A ^a^	B ^b^	C ^c^	D ^d^	E ^e^	F ^f^
Illuminance (one-off record during lab visit)	lux	5587 ± 5501.1 (918)	nm	4208 ± 2560.5 (36)	12316 ± 1823.3 (102)	3682 ± 3246.1 (513)	5877 ± 9262.2 (267)
Temperature (one-off record during lab visit)	°C	25.4 ± 2.12 (603)	20.5 ± 1.25 (13)	24.8 ± 1.17 (48)	25.0 ± 1.89 (102)	26.3 ± 2.32 (204)	25.0 ± 1.83 (236)
Temperature (minimum within a 24-hour period)	°C	20.8 ± 1.97 (75)	nm	nm	20.3 ± 1.87 (8)	21.2 ± 2.02 (33)	20.6 ± 2.05 (34)
Temperature (maximum within a 24-hour period)	°C	26.8 ± 2.59 (75)	nm	nm	25.3 ± 1.98 (8)	27.0 ± 2.83 (33)	26.6 ± 2.26 (34)
Relative humidity (one-off record during lab visit)	%	49 ± 11.7 (488)	nm	nm	42 ± 5.4 (96)	37 ± 7.6 (156)	57 ± 7.8 (236)
Relative humidity (minimum within a 24-hour period)	%	35 ± 7.1 (75)	nm	nm	36 ± 3.7 (8)	30 ± 3.5 (33)	38 ± 8.5 (34)
Relative humidity (maximum within a 24-hour period)	%	55 ± 12.5 (75)	nm	nm	46 ± 5.6 (8)	48 ± 10.5 (33)	63 ± 9.8 (34)

**^a^** A: Overall period (11 October 2013 to 25 September 2014); **^b^** B: Germination period (17 September 2013 to 22 September 2013); **^c^** C: First Planting period (23 September 2013 to 10 October 2013); **^d^** D: Second planting period (11 October 2013 to 7 November 2013); **^e^** E: Final planting period before fruiting (8 November 2013 to 19 January 2014); **^f^** F: Final planting period after fruiting (20 January 2014 to 25 September 2014); nm: not measured.

Figure 3c–f provides summaries of plant developments. Very high numbers of buds were recorded for peppers grown in organic media. Most flowers died before producing any fruits due to the elevated ammonia-nitrogen concentrations supplied to those plants grown in organic media and irrigated by wastewater [43].

Sweet Peppers grown in sand had less buds compared to those peppers grown in organic media. Most buds reached the fruiting stage (Figure 3e), because of a better balance in nutrients supplied to those plants by the irrigation water. The potential of a rather moderate diesel spill to function as stimulation for plant growth in clean water becomes apparent when comparing both controls with each other (Table 1).

Figure 3f summaries a comparison of total weight. Fruits harvested from plants grown in organic media were heavier than those from plants raised in inorganic media. This confirms results obtained by other researchers [17,19] showing the impact of various growth media on pepper harvests.

**Figure 5:**
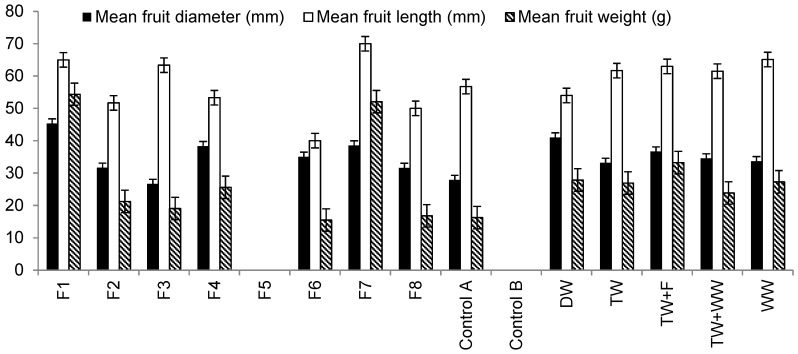
Differences in mean fruit diameter, mean fruit length and mean fruit weight linked to harvested plants (20 January to 25 September 2014) irrigated with different water types and grown in organic media. Notes: No fruit harvest has been noted for plants associated with Filter 5 and Control B.

Figure 5 summaries differences in fruit characteristics. Findings show that fruits harvested from plants irrigated with Filter 1 outflow water were greater than those obtained from peppers irrigated with waters from Filter 2 due to high element loads applied to plants associated with Filter 2 compared to Filter 1 (Table 6). Moreover, fruits belonging to Filter 1 had diameters, which were greater than others indicating the impact of nutrient (mainly nitrogen) and trace element loads provided by irrigation water obtained from Filter 1 compared to the other filters (Table 6).

Figure 5 shows that there is no statistically significant difference in mean fruit length harvested from plants irrigated with different irrigation water types. However, fruits harvested from plants irrigated with Filter 7 (low contact time) outflow water were the longest followed by those irrigated with water obtained from Filters 1 (large aggregate size) and 3 (small aggregate size), which were contaminated with hydrocarbons. The shortest fruit lengths were observed for those harvested from plants irrigated with Filter 6 (high inflow rate) outflow water.

Regarding mean fruit weight, statistical analysis showed that there are significant differences (*p* < 0.05) in fruit mean weight for plants irrigated with water harvested from Filter 6, tap water spiked with fertilizer and raw wastewater. Figure 5 indicates that Sweet Peppers irrigated with water harvested from Filter 1 produced fruits of the highest mean weight (54 g) followed by those harvested from plants irrigated with Filter 7 outflow water, which produced fruits of 52 g mean weight, while the lowest mean fruit weight was recorded for those plants irrigated with Filter 6 outflow water (16 g), explaining the negative impact of high nutrients and trace elements applied to plant fruit weight (Table 6).

**Table 6:** Overview of element mass applied on plants grown in organic media subjected to different irrigation water types (11 October 2013 to 25 September 2014).

Water Type	Total Applied Mass (mg)									
	NH_4_-N ^a^	NO_3_-N ^b^	PO_4_-P ^c^	Ca ^d^	Fe ^e^	K ^f^	Mg ^g^	Mn ^h^	Zn ^i^	B ^j^
Filter 1 outflow	74.8	6.2	62.3	591.9	32.6	149.0	89.0	3.6	1.2	0.8
Filter 2 outflow	87.5	31.0	46.5	484.3	4.6	112.7	71.1	0.9	1.3	0.7
Filter 3 outflow	42.4	4.6	37.8	919.3	24.9	115.1	74.1	4.2	1.4	0.6
Filter 4 outflow	64.5	23.2	37.4	638.7	3.7	50.6	69.8	0.9	1.3	0.5
Filter 5 outflow	107.7	10.0	48.9	834.9	21.8	157.2	104.2	4.2	1.5	0.6
Filter 6 outflow	103.7	41.5	53.0	765.8	11.0	154.1	112.5	1.3	1.7	0.7
Filter 7 outflow	52.2	40.6	52.2	650.8	13.1	105.0	78.9	2.3	1.2	0.4
Filter 8 outflow	15.9	31.8	37.5	506.8	5.4	69.4	57.6	1.0	1.8	0.3
Control A outflow	18.9	5.8	26.1	342.7	3.4	14.5	17.2	1.5	1.5	0.1
Control B outflow	18.3	4.2	26.7	429.8	1.0	11.1	17.8	0.6	2.3	0.1
Deionised water	1.5	<0.1	<0.1	<0.1	1.0	<0.1	1.4	1.2	0.7	<0.1
Tap water (100%)	1.5	10.2	29.7	151.8	49.9	6.2	14.6	0.9	2.7	<0.1
Tap water with fertiliser	276.7	153.9	257.7	179.8	1.6	331.4	20.9	1.6	1.5	<0.1
Wastewater (20%); tap water (80%)	100.0	7.5	44.8	336.6	7.0	51.2	42.1	1.0	3.3	0.1
Wastewater (100%)	580.3	41.4	257.3	1149.9	65.0	244.4	169.1	2.9	4.0	1.0

**^a^** NH_4_-N: ammonia-nitrogen; **^b^** NO_3_-N: nitrate-nitrogen; **^c^** PO_4_-P: ortho-phosphate-phosphorus; **^d^** Ca: calcium; **^e^** Fe: iron; **^f^** K: potassium; **^g^** Mg: magnesium. **^h^** Mn: manganese; **^i^** Zn: zinc; ^j^ B: boron.

Ammonia-nitrogen has a negative effect on plant fruit, leaf and stem developments [15,16]. However, the total yield increases as the nitrate-nitrogen to ammonia-nitrogen ratio increases. This can be explained by a reduction in fruit physiological disorders, which usually reduce fruit mean weight [15,16]. Moreover, high phosphorus levels are known to interfere with the normal metabolism of peppers. Also, it is known to promote manganese uptake by plants [50]. However, researchers [51] reported that high potassium concentration in irrigation water provides protection against stem damage from low night temperatures. Manganese is an essential trace element for most plants, intervening in several metabolic processes (mainly in photosynthesis). Nevertheless, an excess of this micronutrient is often toxic for plants. Manganese phyto-toxicity is exhibited in a reduction of biomass and photosynthesis, and biochemical disorders including oxidative stress [50].

Correlation analysis findings indicated that fruit weights were significantly positively correlated with total water volumes used for irrigation (*R* = 0.821, *p* < 0.001). Since the peppers irrigated with raw wastewater and grown in organic media had the highest number of fruits (Figure 3e), this helps to explain why the total weight of harvested fruits was associated with plants irrigated with raw urban wastewater (Figure 3g). The provision of plants with high nutrient and trace element loads leads to increases in the quantity at the expense of quality of yield.

### 3.4. Sweet Pepper Quality

Table 2 proposes a novel but conservative harvest classification scheme for Sweet Peppers. The lowest variable class entry for any individual pepper fruit assessment determined the final class. If a fruit is categorized, for example, as class A with respect to length, class B in terms of diameter and E regarding weight, then the final class for this fruit is class E. It follows that the corresponding price for this pepper sample will be zero pence (Table 2).

**Figure 6:**
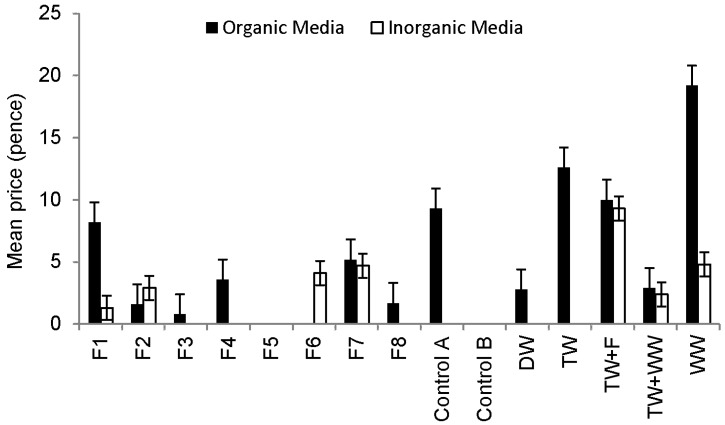
Comparison of the Sweet Pepper harvest outcome linked to plants grown in different media (after classification scheme (Table 2) application).

Figure 6 indicates the monetary value of the pepper harvest. No fruits from any plant were categorized as Class A, B or C. The highest number of fruits categorized as Class D was harvested from peppers grown in organic media and irrigated with raw wastewater followed by those irrigated with tap water, Control A and Filter 1 outflow waters. The highest number of fruits categorized as Class E was harvested also from plants grown in organic media and watered with raw wastewater. No microbial contamination was detected in fruits (skin, flesh and washing solution) harvested from any treatments. However, microbial contamination of peppers is rather unlikely due to the relatively long distance between the fruits and the contaminated soil.

## 4. Conclusions and Further Research

The key research findings have been compared to what was promised in Section 1.4 stating the objectives. Sweet Peppers can be grown using wastewater treated by constructed wetlands. However, the marketable yield was too low to make a decent profit (addressing objective (a)).

The highest number of fruits was linked to raw wastewater and an organic growth medium, while the highest fruit quality indicated by diameter, length and weight was observed for peppers grown in organic media and irrigated with outflow water from wetlands with large aggregates, long contact and resting times, and low inflow loading rate. These results correspond to objectives (b) and (c).

As the nutrients within the degraded compost got depleted about ten months after the start of the experiment, the harvest increased for pots that received pre-treated wastewater in comparison to those depending only on the remaining nutrients obtained from the almost exhausted compost. These findings correspond to objectives (c) and (d).

Results show that nutrient concentrations supplied to the peppers by biodegrading compost and nutrient-rich wastewater were too high to produce a reasonable harvest, because Sweet Peppers are sensitive to too high nutrient concentrations leading to plant development challenges. A high marketable yield (harvest of Sweet Peppers, which are of good quality) related to the most suitable provision of nutrients to the peppers, addressing objectives (c) to (e)).

A good pepper harvest was linked to a wetland system with a large aggregate diameter and diesel spill. This can be explained by the fact that the degradation of hydrocarbon requires the presence of considerable nitrogen resources. In the absence of diesel, too much nitrogen increases leave and decreases fruit developments (addressing objectives (c) to (e)).

Considering that findings indicate the unsuitability for Sweet Peppers to be grown in the tested manner, further research will be undertaken with a mixture of different growth media, and different dilutions of outflow water from wetland filters to optimize the concentrations of nutrients applied to plants supporting the production of the highest yield and best quality. An assessment of the long-term impact of soil and fruit enrichment with minerals will be undertaken in the future. Finally, the authors recommend that field studies should complement experimental studies.
